# Loss of HDAC3 contributes to meiotic defects in aged oocytes

**DOI:** 10.1111/acel.13036

**Published:** 2019-09-09

**Authors:** Yongfu He, Xiaoyan Li, Min Gao, Honglin Liu, Ling Gu

**Affiliations:** ^1^ College of Animal Science & Technology Nanjing Agricultural University Nanjing China

**Keywords:** aneuploidy, HDACs, maternal aging, oocyte quality, reproduction

## Abstract

Maternal age‐related decline in oocyte quality is associated with meiotic defects, but the underlying mechanisms remain to be explored. Histone deacetylase 3 (HDAC3) has been shown to govern multiple cellular events via deacetylating diverse substrates. We previously found that HDAC3 could promote meiotic apparatus assembly in mouse oocytes. In the present study, we identified a substantial reduction in HDAC3 protein in oocytes from old mice. Importantly, overexpression of HDAC3 in old oocytes not only partially prevents spindle/chromosome disorganization, but also significantly lowers the incidence of aneuploidy. Meanwhile, we noticed the elevated acetylation level of α‐tubulin in oocytes derived from old mice. By employing site‐directed mutagenesis, we showed that acetylation‐mimetic mutant tubulin‐K40Q disrupts the kinetochore–microtubule attachments and results in the assembly failure of meiotic apparatus in mouse oocytes. Importantly, forced expression of tubulin‐K40R (nonacetylatable‐mimetic mutant) was capable of alleviating the defective phenotypes of oocytes from aged mice. To sum up, this study uncovers that loss of HDAC3 represents one potential mechanism mediating the effects of advanced maternal age on oocyte quality.

## 1 INTRODUCTION

Advanced maternal age in mammals is associated with reduced fertility. Reproductive capacity in women declines dramatically beyond the mid‐30s (Hamatani et al., 2004; Hassold & Hunt, 2001). In mice, there is an increased frequency of aneuploidy embryos and fetuses with maternal age (Golbus, 1981; Pan, Ma, Zhu, & Schultz, 2008). Emerging evidence suggests that poor oocyte quality is a critical factor mediating the effects of aging on female fertility (Keefe, Kumar, & Kalmbach, 2015; Volarcik et al., 1998). In mammals, the oocytes enter meiosis during the fetal period and their development arrests in the dictyate of prophase until they resume maturation just prior to ovulation at the reproductive age (Kurahashi et al., 2012). It is worth noting that this meiotic process is error prone. Oocytes with the wrong number of chromosomes give rise to aneuploid embryos following fertilization, which in humans is a major cause of pregnancy loss and developmental disabilities (Hassold & Hunt, 2001). Recently, it has been demonstrated that aged oocytes exhibit substantially altered spindle microtubule dynamics, resulting in kinetochore–microtubule attachment defects and chromosome segregation errors (Holubcova, Blayney, Elder, & Schuh, 2015; Nakagawa & FitzHarris, 2017).

Mammalian genomes encode 11 proteins of the classical histone deacetylase family (HDAC1‐11) (Yang & Seto, 2008). These proteins are grouped into class I, II, and IV, with class II being further divided into two subclasses (IIa and IIb), based on their homology to yeast Rpd3/Hda1. HDACs play roles in numerous biological process largely through their influence on transcription by modulating the acetylate state of histones or transcription factors (Haberland, Montgomery, & Olson, 2009). HDAC3, as a member of class I, was found to function in stem cell self‐renewal in a transcription‐independent manner (Li et al., 2006). Accumulated evidence demonstrated that HDAC3 is required for normal mitotic progression through the interaction with Aurora B kinase (Eot‐Houllier, Fulcrand, Watanabe, Magnaghi‐Jaulin, & Jaulin, 2008; Fadri‐Moskwik et al., 2012; Li et al., 2006). Intriguingly, we recently reported that HDAC3 depletion adversely influences meiotic maturation, especially spindle assembly and chromosome organization (Li et al., 2017), similar to the phenotypes of aged oocytes. HDAC3 has been shown to modulate the acetylation state of different substrates in multiple cell lines. For example, in an in vitro reconstituted chromatin system, an HDAC3‐containing protein complex selectively deacetylated histone H3 (Vermeulen et al., 2004). Blocking HDAC3 activity could dramatically alter the tubulin acetylation in the human prostate cancer cells (Bacon et al., 2015). In the present study, we identify a substantial reduction of HDAC3 protein in oocytes from old mice. Notably, overexpression of HDAC3 is capable of alleviating the meiotic defects in oocytes from old mice. These findings indicate that HDAC3 insufficiency in the oocyte may represent a connection between oocyte quality and reproductive aging.

## 2 RESULTS

### 2.1 Reduced HDAC3 expression in oocytes from old mice

**Figure 1:**
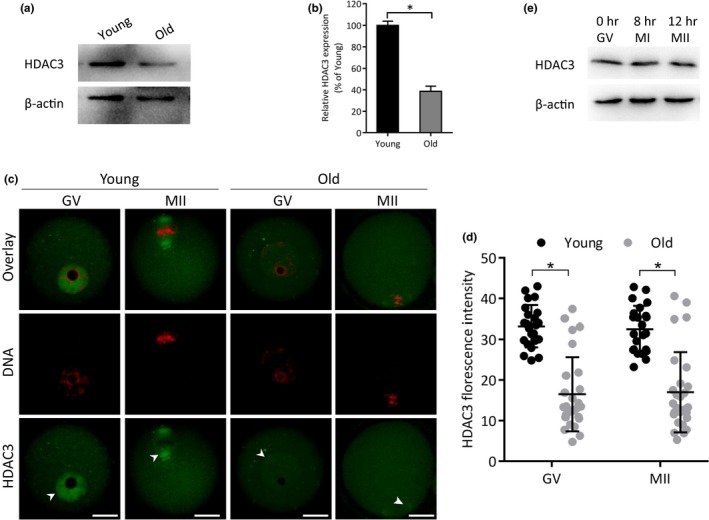
Reduced expression of HDAC3 protein in oocytes from old mice. (a) Representative Western blots showing the HDAC3 expression in oocytes from young and old mice (100 fully grown GV oocytes per lane). (b) Quantification of Western blot results, with actin as a loading control. (c) Confocal sections of young and old oocytes stained with HDAC3 antibody (green) and counterstained with propidium iodide (red) for DNA. Arrowheads indicate the accumulated HDAC3 signal. (d) Quantification of HDAC3 immunofluorescence (*n* = 23 biologically independent oocytes for young; *n* = 27 biologically independent oocytes for old). Scale bars: 20 µm. (e) Representative Western blots showing the HDAC3 expression in oocytes cultured in vitro for 0 h, 8 h, and 12 h.* *p* < .05 versus controls

Given that the oocytes depleted of HDAC3 displayed the similar phenotypes as those from aged mice (Li et al., 2017), we decided to determine the HDAC3 expression in oocytes from young (3 weeks of age) and old (42 weeks of age) mice by Western blot analysis. As shown in Figure 1a‐b, a remarkable reduction of HDAC3 protein was detected in germinal vesicle (GV) stage oocytes from old mice compared to that from young mice. Meanwhile, we also evaluated the HDAC3 expression and distribution in oocytes via immunostaining coupled with confocal microscopy. As shown in Figure 1c‐d, there was no apparent alteration in the distribution pattern of HDAC3 between young and old oocytes. However, the average intensity of HDAC3 signal was significantly decreased in old oocytes relative to young cells. In addition, we found that the levels of HDAC3 protein in different stages of oocytes are comparable when cultured in vitro (Figure 1e), indicating the absence of fluctuation in HDAC3 expression during oocyte maturation.

### 2.2 HDAC3 overexpression alleviates the meiotic defects in old oocytes

**Figure 2:**
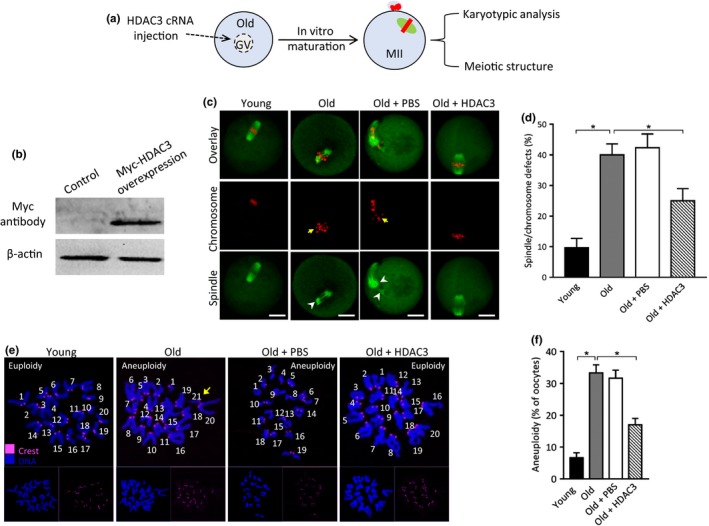
HDAC3 overexpression ameliorates the maternal age‐associated meiotic defects in mouse oocytes. (a) Schematic illustration of the HDAC3 overexpression experiments. (b) Western blotting shows the efficiently overexpressed exogenous HDAC3 protein, probing with anti‐Myc antibody. (c) Young, old, old + PBS, old + HDAC3 oocytes were stained with α‐tubulin antibody to visualize spindle (green) and counterstained with PI to visualize chromosome (red). Arrows point to the misaligned chromosomes and arrowheads indicate the disorganized spindle. Scale bars: 20 µm. (d) Quantification of young (*n* = 116), old (*n* = 110), old + PBS (*n* = 102), old + HDAC3 (*n* = 108) oocytes with spindle/chromosome defects. (e) Chromosome spread of young, old, old + PBS, old + HDAC3 MII oocytes. Chromosomes were stained with Hoechst 33,342 (blue), and kinetochores were labeled with CREST (purple). Arrow points to the extra chromosome in old oocytes. (f) Histogram showing the incidence of aneuploidy in young (*n* = 26), old (*n* = 32), old + PBS (*n* = 30), old + HDAC3 (*n* = 28) oocytes. Error bars indicate ± *SD*. *p < .05 versus controls

To check whether the HDAC3 reduction contributes to the phenotypes of aged oocytes, we conducted the overexpression experiments through the microinjection of cRNA encoding HDAC3 into fully grown old oocytes (Figure 2a). As shown in Figure 2b, immunoblotting verified that exogenous HDAC3 protein was efficiently expressed in oocytes. Spindle/chromosome disorganization was frequently observed in old oocytes (Figure 2c‐d), displaying the scattered chromosomes apart from the metaphase plate. Of note, ectopic expression of HDAC3 ameliorated these meiotic defects in old oocytes. Furthermore, to determine whether overexpression of HDAC3 in old oocytes could also prevent the generation of aneuploid eggs, we analyzed the karyotype of matured oocytes by chromosome spreading. As shown in Figure 2e‐f, maternal aging induced about a threefold increase in incidence of aneuploid eggs compared to controls. Remarkably, the elevated HDAC3 expression in old oocytes significantly decreased the aneuploidy production. Altogether, the results indicate that loss of HDAC3 is one of potential pathways mediating the effects of maternal aging on oocyte quality.

### 2.3 Elevated acetylation levels of tubulin in old oocytes

**Figure 3:**
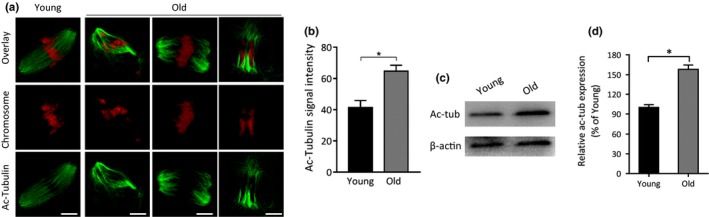
Hyperacetylation of α‐tubulin in old oocytes. (a) Metaphase II oocytes were immunolabeled with acetylated α‐tubulin antibody (green) and counterstained with PI to show chromosomes (red). Representative confocal images of acetylated tubulin in young and old oocytes. (b) Quantification of fluorescence intensity of acetylated tubulin in young (*n* = 17) and old (*n* = 19) oocytes. Error bars indicate ± *SD*. Scale bars: 5 µm. (c) Representative Western blots showing the expression of acetylated tubulin in oocytes from young and old mice. (d) Quantification of Western blot results, with actin as a loading control.* *p* < .05 versus controls

Tubulin is one of the most abundant nonhistone proteins that is subjected to acetylation, which occurs on lysine (K) 40 of α‐tubulin subunit (Zilberman et al., 2009). We previously demonstrated that HDAC3 modulates spindle/chromosome organization in oocytes by maintaining the hypoacetylation state of tubulin (Li et al., 2017). Herein, the reduced HDAC3 expression in old oocytes prompted us to ask whether the acetylation status of tubulin was altered accordingly. To address this question, MII oocytes were isolated from young and old mice. By performing Western blot and immunostaining analysis, we found that the acetylation level of tubulin‐K40 was markedly increased in old oocytes as compared to young oocytes (Figure 3a‐d). This observation implies that tubulin acetylation may be involved in the deficient meiotic apparatus in oocytes from old mice.

### 2.4 Acetylation of tubulin‐K40 functions in kinetochore–microtubule interaction during oocyte meiosis

**Figure 4:**
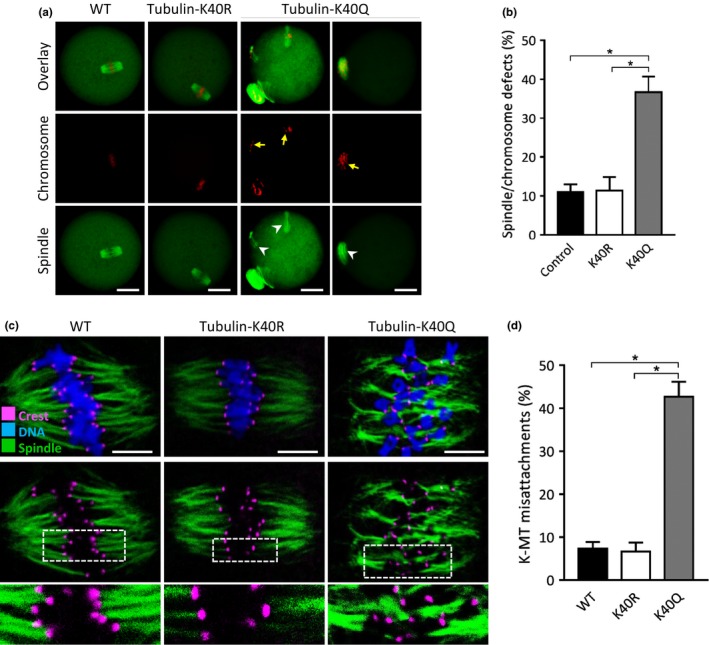
Hyperacetylation of tubulin‐K40 impairs kinetochore–microtubule attachments during oocyte meiosis. (a) WT, WT + K40R, WT + K40Q oocytes were stained with α‐tubulin antibody to visualize spindle (green) and counterstained with PI to visualize chromosome (red). Arrows point to the misaligned chromosomes and arrowheads indicate the disorganized spindle. Scale bars: 20 µm. (b) Quantification of WT, WT + K40R, WT + K40Q oocytes with spindle/chromosome defects. (c) WT, K40Q and K40R mutant cRNA were microinjected into fully grown oocytes for analysis. Metaphase oocytes were stained with CREST for kinetochores (purple), anti‐tubulin antibody for microtubules (green), and Hoechst 33,342 for chromosome (blue). Representative confocal images are shown. Scale bars: 5 µm. (d) Quantitative analysis of K‐MT misattachments in WT (*n* = 16), K40R (*n* = 18), and K40Q (*n* = 13) oocytes. Attachment of kinetochores to microtubules was assessed through examination of the full series of *Z*‐axis focal planes. Kinetochores in regions where fibers were not easily visualized were not included in the analysis. Scale bars: 20 µm. *p < .05 versus controls

To test whether tubulin acetylation affects meiotic structure in oocytes, we constructed the site‐specific mutants (K‐to‐Q and K‐to‐R) targeting lysine 40 (K40) of tubulin, and then the mRNA encoding the tubulin mutants was injected into fully grown oocytes for analysis. As shown in Figure 4a‐b, we found that acetylation‐mimetic mutant K40Q resulted in almost threefold increase in spindle/chromosome defects compared to WT control, whereas K40R had little effects on meiotic apparatus.

Coordination between spindle maintenance and chromosome movement largely relies on kinetochore–microtubule (K‐MT) interaction (Prosser & Pelletier, 2017). Hence, we further examined the effects of tubulin acetylation on K‐MT attachment during meiosis. For this purpose, metaphase oocytes were immunolabeled with CREST to detect kinetochores and stained with anti‐α‐tubulin‐FITC‐conjugated antibody to visualize microtubules. As shown in Figure 4c, the majority of normal oocytes presented amphitelic attachment (each kinetochore attached to one of the poles). However, by performing quantitative analysis (Figure 4d), we found that the proportion of misattachments and loss attachment was significantly increased in oocytes overexpressed K40Q mutant in comparison with K40R oocytes. These erroneous K‐MT attachments would induce unstable chromosome biorientation, causing the abnormal meiotic apparatus in oocytes. Collectively, these results strongly indicate that tubulin‐K40 acetylation controlled by HDAC3 participates in the assembly of meiotic structure in oocytes via maintaining the proper K‐MT interaction.

### 2.5 Tubulin‐K40R mutant ameliorates the meiotic defects in oocytes from old mice

**Figure 5:**
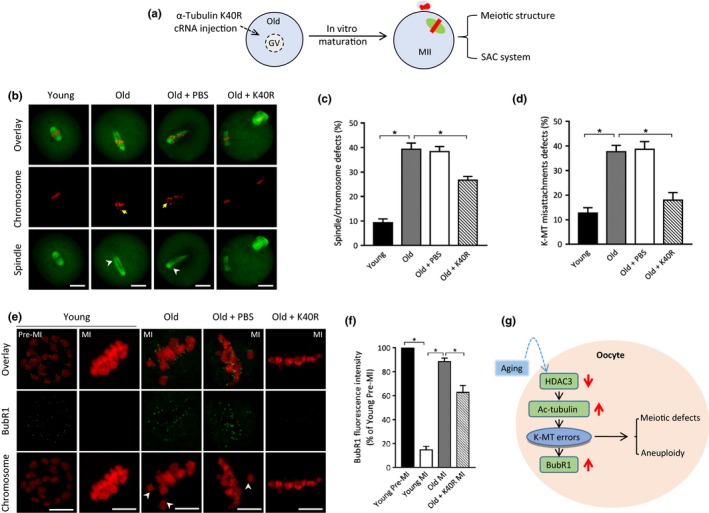
Tubulin‐K40R mutant partly rescues meiotic defects in old oocytes. (a) Schematic illustration of the experimental procedure for overexpression. (b) Young, old, old + PBS, old + Tubulin‐K40R oocytes were stained with α‐tubulin antibody to visualize the spindle (green) and counterstained with PI to visualize chromosomes (red). Arrows point to the misaligned chromosomes and arrowheads indicate the disorganized spindle. Scale bars: 20 µm. (c) Quantification of young, old, old + PBS, old + Tubulin‐K40R oocytes with spindle/chromosome defects. Data are expressed as mean percentage ± *SD* from three independent experiments in which at least 100 oocytes were analyzed. (d) Quantitative analysis of K‐MT misattachments in young (*n* = 20), old (*n* = 22), old + PBS (*n* = 18), old + Tubulin‐K40R (*n* = 20) oocytes. (e) Young, old, old + PBS, old + Tubulin‐K40R oocytes were stained with anti‐BubR1 antibody (green) and counterstained with PI to examine chromosomes (red). Representative confocal images of premetaphase I and metaphase I (MI) oocytes are shown. (f) Quantitative analysis of BubR1 fluorescence intensity in young (*n* = 34), old (*n* = 37), old + PBS (*n* = 29), old + K40R (*n* = 35) oocytes. Error bars indicate ± *SD*. (g) A proposed model showing the potential pathway mediating the effects of aging on oocyte meiosis. Scale bars: 5 µm. *p < .05 versus controls

Given the loss of HDAC3 and hyperacetylation of tubulin in old oocytes, we next checked whether the deacetylation‐mimetic mutant of tubulin could ameliorate their phenotypic defects. To this end, tubulin‐K40R mutant was microinjected into fully grown old oocytes and then in vitro matured for evaluating spindle/chromosome organization (Figure 5a). In comparison with controls, K40R significantly lowered the proportion of abnormal spindle/chromosomes and K‐MT misattachments in old oocytes (Figure 5b‐d). This finding suggests that nonacetylated tubulin‐K40R is capable of partly rescuing the deficient phenotypes of aged oocytes.

Cells have a safety mechanism known as the spindle assembly checkpoint (SAC) to ensure that chromosomes have time to correctly line up on the spindle before the cell can divide (Overlack et al., 2015). Considering the involvement of tubulin acetylation in K‐MT attachments, we further examined whether it affects the SAC system in oocytes. To test this possibility, we analyzed the SAC activity during oocyte meiosis by immunolabeling of BubR1 (budding uninhibited by benzimidazole‐related 1), a core component of the SAC system (Sudakin, Chan, & Yen, 2001). As shown in Figure 5e‐f, BubR1 was localized to the unattached kinetochores at premetaphase I stage in normal oocytes, but was lost when kinetochores are properly attached to microtubules at metaphase I; however, BubR1 signals on the kinetochores in old oocytes at metaphase I stage (MI; culture for 8 hr) were markedly elevated as compared to the counterparts from young mice, indicative of the activation of SAC. Of note, forced expression of tubulin‐K40R mutant in aged oocytes could significantly reduce the BubR1 signals on metaphase chromosomes. Taking together, tubulin acetylation is essential for maintaining the meiotic structure in oocytes probably by modulating the K‐MT‐based SAC system.

## 3 DISCUSSION

Among numerous HDACs, HDAC3 is ubiquitously expressed and conserved in a wide range of species (Ishii, Kurasawa, Wong, & Yu‐Lee, 2008). HDAC3 was found in the nucleus and cytoplasm of different cell types (Longworth & Laimins, 2006; Takami & Nakayama, 2000). It has been reported that HDAC3 could form a well‐characterized protein complex with the NCoR (nuclear receptor co‐repressors) or homologous SMRT (silencing mediator of retinoic and thyroid receptors) to control the transcriptional activity (Bacon et al., 2015; Karagianni & Wong, 2007). Longworth et al. also reported that HDAC3 localizes to the plasma membrane and forms a complex with c‐Src to regulate its activity in HFK + 31 cells (Longworth & Laimins, 2006). In addition, HDAC3 was detected over the spindle in HeLa cells and mouse 3T3 fibroblasts (Ishii et al., 2008). Similarly, we found HDAC3 resides in the cytoplasm of mouse oocytes, with accumulated signals on the meiotic spindle region, promoting the assembly of meiotic apparatus (Li et al., 2017).

It has been well documented that oocytes recovered from old females show a series of changes in multiple nuclear and cytoplasmic structures (Miao, Kikuchi, Sun, & Schatten, 2009). For example, the distribution of cortical granules (CGs) altered dramatically and CG contents became swollen in oocytes from old females (Díaz & Esponda, 2004). Mitochondrial DNA (mtDNA) copy number and function are reduced in eggs from aged mice after both in vivo and in vitro maturation (Pasquariello et al., 2019). In parallel with these observations, Nakagawa et al. found that spindle microtubule dynamics altered substantially in old oocytes, resulting in transiently multipolar spindles that predispose the oocytes to missegregation of intact sister chromatid pairs (Nakagawa & FitzHarris, 2017). However, the underlying molecular details remain to be elucidated. In the present study, we identified a reduction of HDAC3 protein in oocytes from old mice (Figure 1). Importantly, we noted that overexpression of HDAC3 in old oocytes could partially rescue the spindle/chromosomes defects and lower the incidence of aneuploidy eggs (Figure 2). Therefore, these findings support the conclusion that loss of HDAC3 is an important factor contributing to the compromised oocyte quality of aged mice, specifically the failure to assemble the meiotic apparatus.

Microtubules are subject to a remarkable number of post‐translational modifications. Thirty years ago, the conserved ε‐amino group of lysine 40 of the N‐terminal domain of the α‐tubulin was identified as the site of acetylation (LeDizet & Piperno, 1987). The association of acetylation with long‐lived microtubules indicated that stable microtubules tend to be acetylated (Schatten et al., 1988). Reed et al. further showed that loss of α‐tubulin acetylation influences the binding and motility of kinesin‐1 in vitro (Reed et al., 2006). Like other post‐translational modifications of tubulin, acetylation on lysine 40 is under the control of balanced enzyme activities (acetyltransferases and deacetylases). αTAT1 was identified to be the major α‐tubulin acetyltransferase (Kalebic et al., 2013; Shida, Cueva, Xu, Goodman, & Nachury, 2010). Recently, Bacon et al. found that blocking HDAC3 activity modulates tubulin acetylation in the human prostate cancer line PC3 (Bacon et al., 2015). Likewise, we previously showed that the acetylation levels of α‐tubulin were dramatically increased in mouse oocytes depleted of HDAC3 (Li et al., 2017). In line with this observation, here we found the elevated acetylation of tubulin in old oocytes with lowered HDAC3 expression (Figure 3). Moreover, our data showed that nonacetylated tubulin‐K40R is capable of partly rescuing the deficient phenotypes of aged oocytes (Figure 5). These results suggest that HDAC3‐dependent tubulin deacetylation functions as a protective mechanism in oocytes, which is aberrantly blunted in the aged mouse model.

Microtubule stability is regulated in part by a heterogeneous family of proteins that bind to tubulin subunits of microtubules. Related to the functions of microtubules, cyclin B1 interacts with microtubule‐associated proteins (MAPs) to regulate cell cycle progression (Ookata, Hisanaga, Okano, Tachibana, & Kishimoto, 1992). On the other hand, the gradual increase in cyclin B1 levels and CDK1 activity acts as a timing mechanism to modulate the formation of stable K‐MT interaction in oocytes (Davydenko, Schultz, & Lampson, 2013). Herein, we found that the acetylation‐mimetic mutant tubulin‐K40Q induces erroneous K‐MT attachments in oocytes (Figure 5). In combination with the differential meiotic progression between young and old oocytes (Eichenlaub‐Ritter & Boll, 1989), we propose that advanced maternal age induces the loss of HDAC3 in oocytes, likely through elevating tubulin acetylation, results in kinetochore–microtubule misattachments, thereby contributing to the meiotic defects and aneuploidy generation (Figure 5g). In addition, SIRT2 reduction has recently been reported to contribute to oocyte aging by influencing acetylation status of histone H4K16 (Zhang et al., 2014). Therefore, the present study cannot rule out that other pathways might be regulated by HDAC3 to affect meiotic activity in oocytes.

## 4 MATERIALS AND METHODS

All chemicals and culture media were obtained from Sigma unless otherwise specified.

### 4.1 Animals

42‐week‐old female ICR mice were used as a model of reproductive aging, 3‐week‐old female mice were used as a control. All animal protocols were approved by the Animal Care and Use Committee of Nanjing Agricultural University, and all experiments were conducted in accordance with the guidelines of the local animal ethical committee and the Animal Care and Use Committee of Nanjing Agricultural University.

### 4.2 Antibodies

Rabbit polyclonal anti‐HDAC3 antibodies were purchased from Santa Cruz Biotechnology (Cat#: sc‐376957; 1:150); Mouse polyclonal anti‐acetyl‐tubulin (Lys‐40) antibodies were purchased from Sigma (Cat#: T7451; 1:200); Mouse monoclonal FITC‐conjugated anti‐α‐tubulin antibodies were purchased from Sigma (Cat#: F2168; 1:500). Mouse monoclonal anti‐HDAC3 antibodies were purchased from Cell Signaling Technology (Cat#: 3949T; 1:1,000); human anti‐centromere CREST antibody was purchased from Antibodies Incorporated (Davis, CA, USA; Cat#: 15–234; 1:500); Cy5‐conjugated donkey anti‐human IgG and FITC‐conjugated donkey anti‐goat IgG were purchased from Jackson ImmunoResearch Laboratory (Cat#: 709–605–149 and 705–095–147; 1:500); goat polyclonal anti‐BubR1 antibodies and mouse monoclonal anti‐Myc tag antibody were purchased from Abcam (Cat#: ab28193 and ab18185; 1:250); FITC‐conjugated goat anti‐rabbit IgG purchased from Thermo Fisher Scientific (1:300).

### 4.3 Collection and culture of oocytes

Female mice were injected with 5 IU PMSG to stimulate follicular development. Mice were euthanized by cervical dislocation 48 hr after the PMSG priming. Cumulus‐oocyte complexes (COCs) were isolated by rupturing of antral ovarian follicles with sterile needles. Cumulus cells were removed by repeatedly pipetting, and fully grown GV oocytes were cultured in M16 medium under mineral oil at 37°C in a 5% CO2 incubator.

### 4.4 cRNA synthesis and overexpression experiments

Total RNA was extracted from 50 denuded oocytes using the Arcturus PicoPure RNA Isolation Kit (Applied Biosystems), and cDNA was generated using Quantitect Reverse Transcription Kit (Qiagen). The related primers are listed in Table S1. Plasmid construction and mRNA synthesis were conducted as we reported previously (Gao et al., 2018). In brief, PCR products were cloned into the pCS2 + vector with Myc tags, and cRNAs were made using in vitro transcription with SP6 mMESSAGE mMACHINE (Ambion) according to the manufacturer's instruction. Tubulin mutants were generated by site‐directed mutagenesis according to the published protocol (Qiu et al., 2018).

For overexpression experiments, 10 pl cRNA (10 ng/µl) was microinjected into GV oocytes using a micromanipulator (Narishige). The same amount of RNase‐free PBS was injected as a control. After injection, oocytes were cultured in medium containing 2.5 µM milrinone for 20 hr to ensure the translation of cRNA. Following multiple washes, oocytes were cultured in milrinone‐free M16 medium for further experiments.

### 4.5 Immunofluorescence

Oocytes were fixed in 4% paraformaldehyde for 30 min and permeabilized with 0.5% Triton X‐100 for 20 min at room temperature. After blocking in 1% BSA in PBS for 1 hr, oocytes were incubated with primary antibodies overnight at 4°C. Following three washes, oocytes were labeled with secondary FITC‐ or Cy5‐conjugated antibody for 1 hr at room temperature. Samples were mounted on glass slides in a drop of antifade medium (Vectashield) and then examined under a laser scanning confocal microscope (LSM 710). Fluorescent intensity was measured by placing a small circle around the signals using ImageJ software (U.S. National Institutes of Health). The average cytoplasmic fluorescence intensity was subtracted as background.

### 4.6 Chromosome spread

Chromosome spreading was conducted as we previously described (Li et al., 2017). Zona pellucidae was removed prior to fixation by brief exposure to the Tyrode buffer (pH 2.5). Oocytes were washed in M2 medium and then fixed in a drop of 1% paraformaldehyde with 0.15% Triton X‐100 on glass slide. After air drying, a standard immunofluorescent staining procedure was performed as mentioned above. Kinetochores were labeled with CREST and chromosomes were stained with Hoechst 33,342.

### 4.7 Western blotting

A total of 100 GV oocytes were lysed in 2× Laemmli sample buffer per lane. Samples were denatured at 100°C for 5 min, and then frozen at −20°C until use. Lysates were separated by 10% SDS‐PAGE and proteins transferred to PVDF membrane. Membranes were blocked with 5% nonfat milk in Tris‐buffered saline containing 0.1% Tween‐20 (TBST) for 1 hr at room temperature and then probed with primary antibodies (Myc antibody, 1:1,000; HDAC3 antibody, 1:1,000) at 4°C overnight. After multiple washes in TBST and incubation with HRP‐conjugated secondary antibodies, the protein bands were visualized using an ECL Plus Western Blotting Detection System. β‐actin was used as a loading control.

### 4.8 Statistical analysis

Experiments were performed at least in triplicate. Data are presented as means ± *SD*, unless otherwise stated. Differences between two groups were analyzed by Student's *t* test. Multiple comparisons between more than two groups were analyzed by one‐way ANOVA test using Prism 5.0. *p* < .05 was considered to be significant.

## CONFLICT OF INTEREST

The authors have nothing to disclose.

## AUTHOR CONTRIBUTIONS

Y.H. and L.G. designed research; Y.H., X.L., M.G. performed research; L.G. and H.L. analyzed data; Y.H. and L.G. wrote paper.

## References

[acel13036-bib-0001] Bacon, T. , Seiler, C. , Wolny, M. , Hughes, R. , Watson, P. , Schwabe, J. , … Peckham, M. (2015). Histone deacetylase 3 indirectly modulates tubulin acetylation. The Biochemical Journal, 472, 367–377. 10.1042/BJ20150660 26450925 PMC4661566

[acel13036-bib-0002] Davydenko, O. , Schultz, R. M. , & Lampson, M. A. (2013). Increased CDK1 activity determines the timing of kinetochore‐microtubule attachments in meiosis I. Journal of Cell Biology, 202, 221–229. 10.1083/jcb.201303019 23857768 PMC3718970

[acel13036-bib-0003] Díaz, H. , & Esponda, P. (2004). Ageing‐induced changes in the cortical granules of mouse eggs. Zygote, 12, 95–103. 10.1017/S0967199404002680 15460103

[acel13036-bib-0004] Eichenlaub‐Ritter, U. , & Boll, I. (1989). Nocodazole sensitivity, age‐related aneuploidy, and alterations in the cell cycle during maturation of mouse oocytes. Cytogenetics and Cell Genetics, 52, 170–176. 10.1159/000132871 2535312

[acel13036-bib-0005] Eot‐Houllier, G. , Fulcrand, G. , Watanabe, Y. , Magnaghi‐Jaulin, L. , & Jaulin, C. (2008). Histone deacetylase 3 is required for centromeric H3K4 deacetylation and sister chromatid cohesion. Genes & Development., 22, 2639–2644. 10.1101/gad.484108 18832068 PMC2559902

[acel13036-bib-0006] Fadri‐Moskwik, M. , Weiderhold, K. N. , Deeraksa, A. , Chuang, C. , Pan, J. , Lin, S. H. , & Yu‐Lee, L. Y. (2012). Aurora B is regulated by acetylation/deacetylation during mitosis in prostate cancer cells. The FASEB Journal, 26, 4057–4067. 10.1096/fj.12-206656 22751009 PMC3448774

[acel13036-bib-0007] Gao, M. , Li, X. , He, Y. , Han, L. , Qiu, D. , Ling, L. , … Gu, L. (2018). SIRT7 functions in redox homeostasis and cytoskeletal organization during oocyte maturation. The FASEB Journal, fj201800078RR 10.1096/fj.201800078RR 29879377

[acel13036-bib-0008] Golbus, M. S. (1981). The influence of strain, maternal age, and method of maturation on mouse oocyte aneuploidy. Cytogenetic and Genome Research, 31, 84–90. 10.1159/000131629 7198025

[acel13036-bib-0009] Haberland, M. , Montgomery, R. L. , & Olson, E. N. (2009). The many roles of histone deacetylases in development and physiology: Implications for disease and therapy. Nature Reviews Genetics, 10, 32–42. 10.1038/nrg2485 PMC3215088 19065135

[acel13036-bib-0010] Hamatani, T. , Falco, G. , Carter, M. G. , Akutsu, H. , Stagg, C. A. , Sharov, A. A. , … Ko, M. S. (2004). Age‐associated alteration of gene expression patterns in mouse oocytes. Human Molecular Genetics, 13, 2263–2278. 10.1093/hmg/ddh241 15317747

[acel13036-bib-0011] Hassold, T. , & Hunt, P. (2001). To err (meiotically) is human: The genesis of human aneuploidy. Nature Reviews Genetics, 2, 280–291. 10.1038/35066065 11283700

[acel13036-bib-0012] Holubcova, Z. , Blayney, M. , Elder, K. , & Schuh, M. (2015). Human oocytes. Error‐prone chromosome‐mediated spindle assembly favors chromosome segregation defects in human oocytes. Science, 348, 1143–1147. 26045437 10.1126/science.aaa9529 PMC4477045

[acel13036-bib-0013] Ishii, S. , Kurasawa, Y. , Wong, J. , & Yu‐Lee, L. Y. (2008). Histone deacetylase 3 localizes to the mitotic spindle and is required for kinetochore‐microtubule attachment. Proceedings of the National Academy of Sciences of the United States of America, 105, 4179–4184. 10.1073/pnas.0710140105 18326024 PMC2393771

[acel13036-bib-0014] Kalebic, N. , Sorrentino, S. , Perlas, E. , Bolasco, G. , Martinez, C. , & Heppenstall, P. A. (2013). alphaTAT1 is the major alpha‐tubulin acetyltransferase in mice. Nature Communications, 4, 1962. 10.1038/ncomms2962 23748901

[acel13036-bib-0015] Karagianni, P. , & Wong, J. (2007). HDAC3: Taking the SMRT‐N‐CoRrect road to repression. Oncogene, 26, 5439–5449. 10.1038/sj.onc.1210612 17694085

[acel13036-bib-0016] Keefe, D. , Kumar, M. , & Kalmbach, K. (2015). Oocyte competency is the key to embryo potential. Fertility and Sterility, 103, 317–322. 10.1016/j.fertnstert.2014.12.115 25639967

[acel13036-bib-0017] Kurahashi, H. , Tsutsumi, M. , Nishiyama, S. , Kogo, H. , Inagaki, H. , & Ohye, T. (2012). Molecular basis of maternal age‐related increase in oocyte aneuploidy. Congenit Anom (Kyoto), 52, 8–15. 10.1111/j.1741-4520.2011.00350.x 22348779

[acel13036-bib-0018] LeDizet, M. , & Piperno, G. (1987). Identification of an acetylation site of Chlamydomonas alpha‐tubulin. Proceedings of the National Academy of Sciences of the United States of America, 84, 5720–5724. 10.1073/pnas.84.16.5720 2441392 PMC298934

[acel13036-bib-0019] Li, X. , Liu, X. , Gao, M. , Han, L. , Qiu, D. , Wang, H. , … Gu, L. (2017). HDAC3 promotes meiotic apparatus assembly in mouse oocytes by modulating tubulin acetylation. Development, 144, 3789–3797. 10.1242/dev.153353 28935703

[acel13036-bib-0020] Li, Y. , Kao, G. D. , Garcia, B. A. , Shabanowitz, J. , Hunt, D. F. , Qin, J. , … Lazar, M. A. (2006). A novel histone deacetylase pathway regulates mitosis by modulating Aurora B kinase activity. Genes & Development., 20, 2566–2579. 10.1101/gad.1455006 16980585 PMC1578679

[acel13036-bib-0021] Longworth, M. S. , & Laimins, L. A. (2006). Histone deacetylase 3 localizes to the plasma membrane and is a substrate of Src. Oncogene, 25, 4495–4500. 16532030 10.1038/sj.onc.1209473

[acel13036-bib-0022] Miao, Y. L. , Kikuchi, K. , Sun, Q. Y. , & Schatten, H. (2009). Oocyte aging: Cellular and molecular changes, developmental potential and reversal possibility. Human Reproduction Update, 15, 573–585. 10.1093/humupd/dmp014 19429634

[acel13036-bib-0023] Nakagawa, S. , & FitzHarris, G. (2017). Intrinsically defective microtubule dynamics contribute to age‐related chromosome segregation errors in mouse oocyte meiosis‐I. Current Biology, 27, 1040–1047. 10.1016/j.cub.2017.02.025 28376326

[acel13036-bib-0024] Ookata, K. , Hisanaga, S. , Okano, T. , Tachibana, K. , & Kishimoto, T. (1992). Relocation and distinct subcellular localization of p34cdc2‐cyclin B complex at meiosis reinitiation in starfish oocytes. The EMBO Journal, 11, 1763–1772. 10.1002/j.1460-2075.1992.tb05228.x 1316272 PMC556634

[acel13036-bib-0025] Overlack, K. , Primorac, I. , Vleugel, M. , Krenn, V. , Maffini, S. , Hoffmann, I. , … Musacchio, A. (2015). A molecular basis for the differential roles of Bub1 and BubR1 in the spindle assembly checkpoint. eLife, 4, e05269 10.7554/eLife.05269 25611342 PMC4337726

[acel13036-bib-0026] Pan, H. , Ma, P. , Zhu, W. , & Schultz, R. M. (2008). Age‐associated increase in aneuploidy and changes in gene expression in mouse eggs. Developmental Biology, 316, 397–407. 10.1016/j.ydbio.2008.01.048 18342300 PMC2374949

[acel13036-bib-0027] Pasquariello, R. , Ermisch, A. F. , Silva, E. , McCormick, S. , Logsdon, D. , Barfield, J. P. , … Krisher, R. L. (2019). Alterations in oocyte mitochondrial number and function are related to spindle defects and occur with maternal aging in mice and humans. Biology of Reproduction, 100, 971–981. 30476005 10.1093/biolre/ioy248

[acel13036-bib-0028] Prosser, S. L. , & Pelletier, L. (2017). Mitotic spindle assembly in animal cells: A fine balancing act. Nature Reviews Molecular Cell Biology, 18, 187–201. 28174430 10.1038/nrm.2016.162

[acel13036-bib-0029] Qiu, D. , Hou, X. , Han, L. , Li, X. , Ge, J. , & Wang, Q. (2018). Sirt2‐BubR1 acetylation pathway mediates the effects of advanced maternal age on oocyte quality. Aging Cell, 17 10.1111/acel.12698 PMC5770883 29067790

[acel13036-bib-0030] Reed, N. A. , Cai, D. , Blasius, T. L. , Jih, G. T. , Meyhofer, E. , Gaertig, J. , & Verhey, K. J. (2006). Microtubule acetylation promotes kinesin‐1 binding and transport. Current Biology, 16, 2166–2172. 10.1016/j.cub.2006.09.014 17084703

[acel13036-bib-0031] Schatten, G. , Simerly, C. , Asai, D. J. , Szoke, E. , Cooke, P. , & Schatten, H. (1988). Acetylated alpha‐tubulin in microtubules during mouse fertilization and early development. Developmental Biology, 130, 74–86. 3053299 10.1016/0012-1606(88)90415-0

[acel13036-bib-0032] Shida, T. , Cueva, J. G. , Xu, Z. , Goodman, M. B. , & Nachury, M. V. (2010). The major alpha‐tubulin K40 acetyltransferase alphaTAT1 promotes rapid ciliogenesis and efficient mechanosensation. Proceedings of the National Academy of Sciences of the United States of America, 107, 21517–21522. 21068373 10.1073/pnas.1013728107 PMC3003046

[acel13036-bib-0033] Sudakin, V. , Chan, G. K. , & Yen, T. J. (2001). Checkpoint inhibition of the APC/C in HeLa cells is mediated by a complex of BUBR1, BUB3, CDC20, and MAD2. Journal of Cell Biology, 154, 925–936. 10.1083/jcb.200102093 11535616 PMC2196190

[acel13036-bib-0034] Takami, Y. , & Nakayama, T. (2000). N‐terminal region, C‐terminal region, nuclear export signal, and deacetylation activity of histone deacetylase‐3 are essential for the viability of the DT40 chicken B cell line. The Journal of Biological Chemistry, 275, 16191–16201. 10.1074/jbc.M908066199 10748092

[acel13036-bib-0035] Vermeulen, M. , Carrozza, M. J. , Lasonder, E. , Workman, J. L. , Logie, C. , & Stunnenberg, H. G. (2004). In vitro targeting reveals intrinsic histone tail specificity of the Sin3/histone deacetylase and N‐CoR/SMRT corepressor complexes. Molecular and Cellular Biology, 24, 2364–2372. 10.1128/MCB.24.6.2364-2372.2004 14993276 PMC355843

[acel13036-bib-0036] Volarcik, K. , Sheean, L. , Goldfarb, J. , Woods, L. , Abdul‐Karim, F. W. , & Hunt, P. (1998). The meiotic competence of in‐vitro matured human oocytes is influenced by donor age: Evidence that folliculogenesis is compromised in the reproductively aged ovary. Human Reproduction (Oxford, England), 13, 154–160. 10.1093/humrep/13.1.154 9512249

[acel13036-bib-0037] Yang, X. J. , & Seto, E. (2008). The Rpd3/Hda1 family of lysine deacetylases: From bacteria and yeast to mice and men. Nature Reviews Molecular Cell Biology, 9, 206–218. 18292778 10.1038/nrm2346 PMC2667380

[acel13036-bib-0038] Zhang, L. , Hou, X. , Ma, R. , Moley, K. , Schedl, T. , & Wang, Q. (2014). Sirt2 functions in spindle organization and chromosome alignment in mouse oocyte meiosis. The FASEB Journal, 28, 1435–1445. 10.1096/fj.13-244111 24334550 PMC3929683

[acel13036-bib-0039] Zilberman, Y. , Ballestrem, C. , Carramusa, L. , Mazitschek, R. , Khochbin, S. , & Bershadsky, A. (2009). Regulation of microtubule dynamics by inhibition of the tubulin deacetylase HDAC6. Journal of Cell Science, 122, 3531–3541. 10.1242/jcs.046813 19737819

