# Supplementary material for: Loss of HDAC3 contributes to meiotic defects in aged oocytes
Source: Aging Cell. 2019 Sep 9;18(6):e13036. doi: 10.1111/acel.13036 (PMC6826132; doi:10.1111/acel.13036)
Supplement: Supplementary file 1 [file ACEL-18-e13036-s001.docx]

**Table S1** Primer sequences of genes for cDNA amplification and mutant construction

***Gene*** ***Primer sequence***

HDAC3

Forward primer: 5′-GGGGGCCGGCCCCATGGCCAAGACCGTGG-3′

Reverse primer:5′-GGGGGCGCGCCGGGGAAGAGGGGCTGAGG-3′

α-Tubulin

Forward primer:5′-GGG GGCCGGCCATGCGTGAGTGCATCTCC -3′

Reverse primer:5′-GGGGGCGCGCCCACAATAAACATCCCTGTGG-3′

Tubulin-K40R

Forward primer:5′-CAGATGCCAAGTGACAGGACCATTGGGGGA-3′

Reverse primer: 5′-CTGTCACTTGGCATCTGGCCATCAGGC-3′

Tubulin-K40Q

Forward primer:5′-CAGATGCCAAGTGACCAGACCATTGGGGGA-3′

Reverse primer: 5′-GGTCACTTGGCATCTGGCCATCAGGC-3′
